# Design Choices and Trade-Offs in Health Care Blockchain Implementations: Systematic Review

**DOI:** 10.2196/12426

**Published:** 2019-05-10

**Authors:** Odhran O'Donoghue, Anuraag A Vazirani, David Brindley, Edward Meinert

**Affiliations:** 1 Medical School Medical Sciences Division University of Oxford Oxford United Kingdom; 2 Healthcare Translation Research Group Department of Paediatrics University of Oxford Oxford United Kingdom; 3 Global Digital Health Unit Department of Primary Care and Public Health Imperial College London London United Kingdom

**Keywords:** blockchain, interoperability, distributed ledger technology, scalability, health information exchange

## Abstract

**Background:**

A blockchain is a list of records that uses cryptography to make stored data immutable; their use has recently been proposed for electronic medical record (EMR) systems. This paper details a systematic review of trade-offs in blockchain technologies that are relevant to EMRs. Trade-offs are defined as “a compromise between two desirable but incompatible features.”

**Objective:**

This review’s primary research question was: “What are the trade-offs involved in different blockchain designs that are relevant to the creation of blockchain-based electronic medical records systems?”

**Methods:**

Seven databases were systematically searched for relevant articles using the Preferred Reporting Items for Systematic Reviews and Meta-Analyses (PRISMA). Papers published from January 1, 2017 to June 15, 2018 were selected. Quality assessments of papers were performed using the Risk Of Bias In Non-randomized Studies—of Interventions (ROBINS-I) tool and the Critical Assessment Skills Programme (CASP) tool. Database searches identified 2885 articles, of which 15 were ultimately included for analysis.

**Results:**

A total of 17 trade-offs were identified impacting the design, development, and implementation of blockchain systems; these trade-offs are organized into themes, including business, application, data, and technology architecture.

**Conclusions:**

The key findings concluded the following: (1) multiple trade-offs can be managed adaptively to improve EMR utility; (2) multiple trade-offs involve improving the security of blockchain systems at the cost of other features, meaning EMR efficacy highly depends on data protection standards; and (3) multiple trade-offs result in improved blockchain scalability. Consideration of these trade-offs will be important to the specific environment in which electronic medical records are being developed. This review also uses its findings to suggest useful design choices for a hypothetical National Health Service blockchain.

**International Registered Report Identifier (IRRID):**

RR2-10.2196/10994

## Introduction

### Background

Blockchains contain *blocks* of data ordered chronologically. Each block is linked to previous blocks via cryptography. If previous blocks are edited, these cryptographic linkers will no longer match, making blockchains resistant to tampering. Blockchains originated in 1991 [1], but the first major adoption was 2008’s Bitcoin [2]. For Bitcoin’s creator, the use case for blockchains is to create data ledgers that can be trusted without centralized systems: updates to its blockchain have to be agreed on by its users through consensus processes. Bitcoin’s use of blockchain works as described in the following sections.

### Block Creation

**Figure 1:**
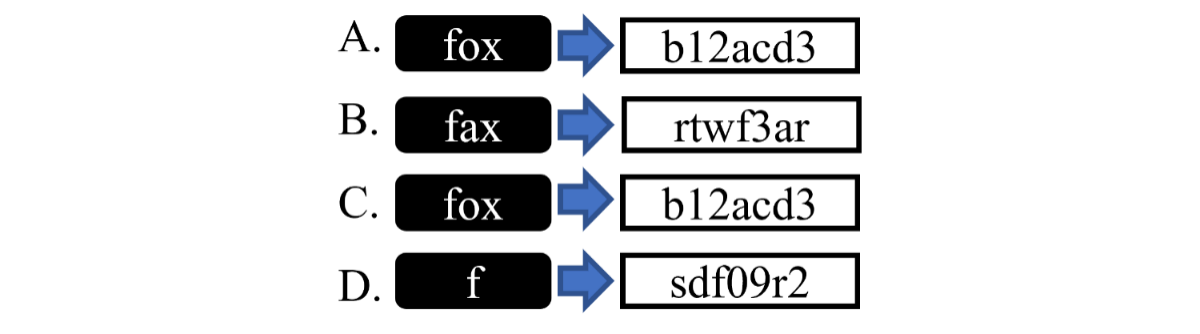
A: hash functions convert data into fixed-length strings (hashes). B: similar but unidentical data have very different hashes. C: identical data have identical hashes, allowing for verification. D: all lengths of data produce fixed-length hashes.

**Figure 2:**
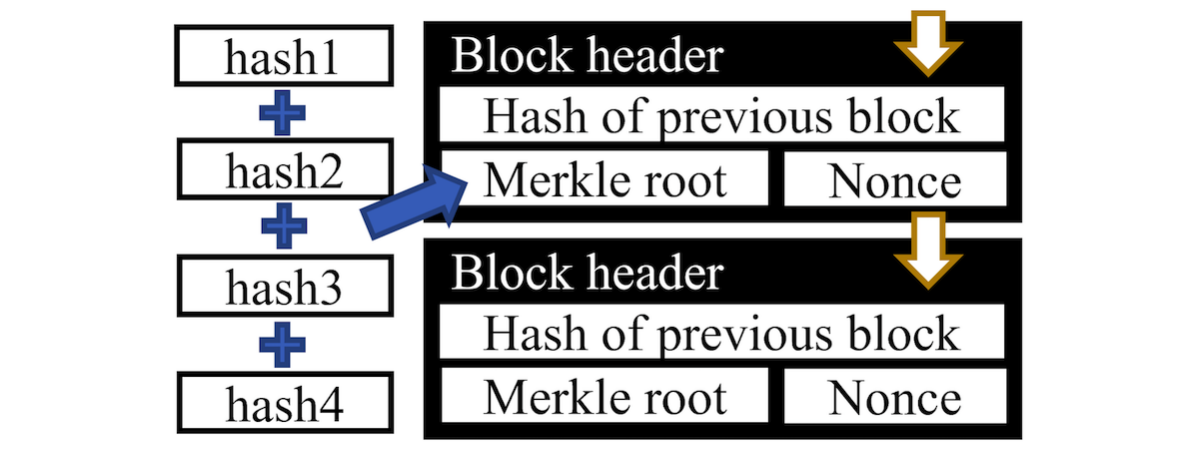
The hash of the previous block, the Merkle root, and the Nonce are combined into a single hash. This is included in the next block header, linking the two blocks.

Transactions between individuals are recorded. These data are *encrypted* via hash functions [3], which convert data into alphanumeric strings known as hashes (see Figure 1). The data inputted cannot be decrypted from these hashes. Each transaction is hashed and a summary hash, known as a *Merkle root*, is generated for each block of data [4] (see Figure 2).

### Adding a New Block to the Blockchain

To add new blocks, a majority of users must want the same new block (ie, consensus). However, malicious users could create multiple accounts to gain control. To avoid this, Bitcoin uses proof of work (PoW); this requires the users’ processing power for consensus, which will not increase for a user owning multiple accounts. PoW works when the network randomly generates a *target hash*. To add a block to the chain, users must find a number that, when added to the Merkle root and hashed, equals the target hash. This is known as *mining*.

Mining is computationally difficult but finding the right number (ie, a nonce) is rewarded with bitcoins. If miners use their combined computational power to mine the same block and share the rewards after finding the nonce, they are more likely to make money than if mining alone. This encourages consensus.

A new block is formed on the chain when a correct nonce is found. The hashes of the previous block are combined into the Merkle root of the new block, cryptographically linking the two blocks (see Figure 1).

### Blockchain Security

Were someone to try to maliciously edit a transaction, they would have to mine a new nonce. This is because a different transaction value needs a different nonce to get the target hash. Furthermore, edits would need to be made to subsequent blocks to ensure chain coherence. For malicious users to find nonces faster than the rate at which nonces are found on the *true* chain is not feasible without obtaining majority control of a network: this is known as a 51% attack [5].

### The Use of Blockchain as an Electronic Medical Record

As described in the 2019 Topol Review [6] and the 2019 National Health Service (NHS) Long-Term Plan [7], there is a drive to adopt electronic medical records (EMRs) throughout the NHS. The reports posit that such systems are needed for personalized long-term care and for obtaining the large-scale datasets necessary for predictive health modelling. Current strategic objectives are to operate multiple EMRs that are interoperable through standards laid out in the Local Health and Care Record Exemplars [8]. Blockchains have been proposed as an EMR platform and could be considered by the NHS as they may have some advantages over classical EMRs: distributed ledgers require no manual reconciliation of data between different providers [9]. This is a common issue with current EMRs, as patients can encounter many health care providers. Secondly, the ledger includes an audit trail of all changes. This helps to ensure EMR integrity and prevent data falsification [9]. Distributed systems would also allow interoperability to shift from a provider-driven model to a patient-driven model [10], in which patients are empowered to add to and exchange their own health data. An example of a blockchain EMR is MedRec [11], which is built on the Ethereum blockchain. Ethereum supports *smart contracts*, which are codes that self-execute when certain criteria are met [12]. MedRec only stores metadata on the blockchain and uses smart contracts to let certified users access full EMR data stored off-chain. This improves scalability, but a trade-off is that EMR data may be harder to audit.

### Trade-Offs in Blockchain Design

While the principle of cryptographically linked blocks underpins a blockchain, many design choices can be made [13]. However, these choices may result in trade-offs [14]: trade-offs are defined as “a compromise between two desirable but incompatible features.” An example of where a trade-off has been made in the design of a blockchain-based EMR is in the creation of version 2.0 of MedRec, where the amount of information stored on the MedRec blockchain was reduced to improve scalability. Understanding the potential trade-offs in blockchain design is important, as it better informs the development of blockchain-based EMRs. Historic failings in the adoption of UK EMRs [15] indicate that new EMR technology should be investigated thoroughly before adoption. Therefore, how design trade-offs affect the suitability of a blockchain for EMR use should be explored. This review aims to improve the understanding of NHS clinicians and policy makers interested in adopting a blockchain EMR and to inform blockchain developers of design trade-offs relevant to EMRs.

## Methods

### Formulation of Research Question

This review’s research question was derived from Meinert et al’s review protocol [16]. In comparison to Meinert et al’s protocol, this review’s research question has three modifications: (1) to prevent the scope of the review from becoming too broad, the research question only considers blockchain design and does not consider blockchain implementation strategies and frameworks; (2) the review’s research question specifically focuses on trade-offs in blockchain design, as a research deficit in this area has been identified [14]; and (3) the review’s research question does not limit research domains to only privacy, efficiency, interoperability, and scalability.

### Research Question

What are the trade-offs involved in different blockchain designs that are relevant to the creation of blockchain-based electronic medical records systems? This research question is based on the following definitions, which are sorted into Population, Intervention, Comparison, and Outcome (PICO) criteria (see Table 1).

### Search Strategy and Inclusion Criteria

**Figure 3:**
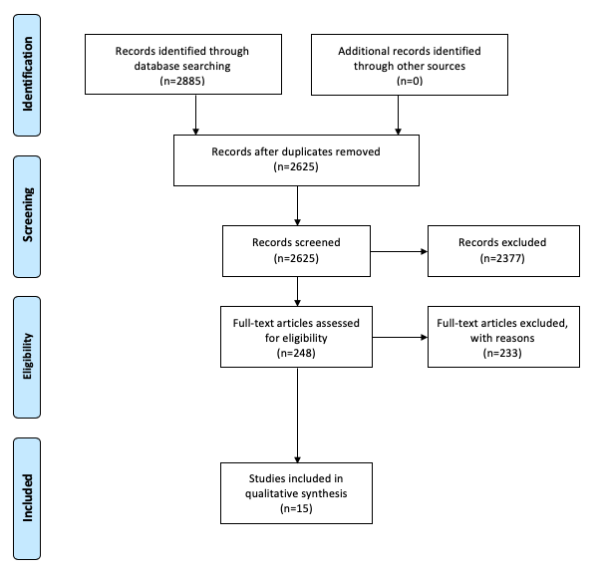
Preferred Reporting Items for Systematic Reviews and Meta-Analyses (PRISMA) flow diagram of the search strategy.

The selection process of this review is demonstrated using the Preferred Reporting Items for Systematic Reviews and Meta-Analyses (PRISMA) flow diagram (see Figure 3). PubMed, MEDLINE, Embase, Web of Science, and Scopus databases were searched. Papers published from January 1, 2017 to June 15, 2018 were selected. Search string creation (see Multimedia Appendix 1) was aided by the Nuffield Orthopaedic Centre Library. Inclusion criteria for database searches were based on PICO criteria (see Table 1). “Blockchain” (*Population*) was used in all search strings. This was combined with the term “trade-off” (*Outcome*) and variants thereof. “Blockchain” was then combined with “design” (*Intervention*) and variants thereof. “Blockchain” was also combined with “Electronic medical record” (*Comparison*) and variants thereof. Variants were found, in part, with Medical Subject Headings (MeSH) terms. Screening criteria based on key phrases were used to remove papers (see Table 2). Abstracts of remaining papers were screened to determine suitability. The PICO criteria formed the paper inclusion criteria. If abstracts contained PICO criteria or did not contain sufﬁcient information, articles were read in full. A total of 90 articles were read in full to determine suitability. Following peer review from Institute of Electrical and Electronics Engineers (IEEE) Access, a search of Springer Link and IEEE databases was requested to find more nonmedical perspectives on blockchain design. Papers were searched that were published between January 1, 2017 and December 5, 2018. In this second search, 158 articles were read in full to determine suitability. Due to time limits, only papers written before the initial June 15, 2018 deadline were included in the qualitative synthesis. A total of 2885 papers were identified. After screening and duplicate removal, 248 articles remained, all of which were assessed for eligibility by analyzing their full text. A total of 233 articles were removed: the reasons for removal are outlined in Figure 3.

**Table 1:** PICO (Population, Intervention, Comparison, and Outcome) criteria, terms, and definitions.

PICO criterion	Term	Definition
Population	Blockchain	A growing list of records, called blocks, which are linked using cryptography
Intervention	Design	A decision about object function with a specific purpose in mind
Comparison	Electronic medical record	A systematized digital collection and storage of patient data
Outcome	Trade-off	A compromise between two desirable but incompatible features

**Table 2:** Screening criteria used to remove papers.

Criterion number	Screening criteria	Justification	Removed in pre-peer-review search	Removed in post-peer-review search
1	Papers not containing abstracts	The papers cannot be analyzed at the abstract-screening stage and were therefore excluded.	75	0
2	Papers from 2013-2016	As an emerging technology, ideas about blockchain’s possibilities and limitations are often changing. Older papers were not included, as they may contain information that does not reflect the current state of blockchain technologies. Papers before 2017 were chosen to be excluded, as 2017 was a year characterized by a significant change in blockchain valuation and regulation [26].	328	52
3	Full conference proceedings	The relevant full papers in the conference proceedings should have been identified as individual papers in database searches and included in the 1808 papers.	63	0
4	Duplicate titles	These articles contain repeated information.	125	2
5	Does not contain “block” in abstract	Articles that do not mention “block” in their abstract were deemed unlikely to be focused discussions of blockchain technologies and were therefore not included.	419	89
6	Does not contain “design” in abstract	As a PICO^a^ term, a focus on blockchain design was essential to the papers. No alternative MeSH^b^ terms for “design” were identified, so an absence of “design” in abstracts was used to filter out papers.	590	486
7	Title contains “Bitcoin” and not “block”	Papers that mentioned “Bitcoin” in their title and not “blockchain” were deemed to be too focused on the cryptocurrency to provide useful information concerning principles relevant to blockchain design.	12	6
8	Title contains “IoT” or “Internet of things”	Many papers contained a reference to IoT^c^ devices. The abstracts of these papers contained little-to-no relevant information about blockchains. This exclusion criterion was thus used to remove papers.	15	24
9	Title contains “finance”	It was a concern that papers relating to financial elements of blockchains would be too focused on the economic aspects of cryptocurrency. As the intent of this research is to examine the technical, not economical, aspects of blockchains, these papers were removed.	15	2

^a^PICO: Population, Intervention, Comparison, and Outcome.

^b^MeSH: Medical Subject Headings.

^c^IoT: internet of things.

### Assessment of Methodological Quality

The Risk Of Bias In Non-randomized Studies—of Interventions (ROBINS-I) framework was used to assess bias in the 15 remaining papers [17] (see Multimedia Appendix 2). For eight papers lacking experimental data, ROBINS-I was deemed unsuitable, and a qualitative medical data assessment tool developed by the Critical Assessment Skills Programme (CASP) was used instead [18] (see Multimedia Appendix 3). ROBINS-I identified a low risk of bias in six papers [19-24] and a serious risk of bias in one paper [25]: this paper was removed. Thus, trade-offs in 14 papers are discussed.

### Data Synthesis

Only narrative data synthesis was appropriate due to study heterogeneity. To structure this, an enterprise architecture framework (ie, a set of principles used to guide enterprise design, planning, and implementation) was used. The Open Group Architecture Framework (TOGAF) [27] was chosen, as the framework is in use by software industry leaders [28]. TOGAF divides enterprise architecture into four domains: Technology (ie, the hardware underpinning programs), Data (ie, choices made in data formatting), Application (ie, software applications and procedures), and Business (ie, decisions about the business structure that relate to enterprise architecture development).

## Results

### Technology Architecture Trade-Offs

#### Current Implementation Versus Future Proofing

A critical technology architecture trade-off was current implementation versus future proofing [29]. The advent of quantum computing may compromise current blockchain cryptography. Quantum-resistant cryptography is being developed—lattice problems are being considered for this role [30]—but research is still ongoing. This is a trade-off for hospital systems to consider: to invest in blockchain systems designed for today’s computers or to wait until quantum-resistant cryptography is developed. Such a choice will depend on a health care provider’s ability to update its system. If the provider is large and decentralized, it may struggle to simultaneously switch over to a new blockchain; initially investing in a postquantum blockchain may be more sensible, even if it delays implementation.

#### Stale Block Rate Versus Network Delay

Another trade-off is stale block rate versus network delay [23]. At any time, a miner may successfully find a nonce and broadcast it across the network so that miners can begin working on the next block. Delays in the network result in miners wasting computational power by continuing to mine a block that has already been solved, which is known as a *stale block*. Simulations in a study by Chen et al [23] show that the stale block rate correlates with network delay. Reducing delay involves increasing network infrastructure provision. Thus, decisions about the network infrastructure provided for EMRs will impact blockchain performance. An alternative detailed below is to increase the block window, but this also has trade-offs.

### Data Architecture Trade-Offs

#### Block Window: Computation Versus Communication

One data architecture trade-off is block window: computation versus communication [31]. Block window is a measure of how many blocks are created per minute. Smaller windows allow for faster transaction confirmation but more network bandwidth is required, as a larger number of blocks is broadcast through the network. While larger windows will reduce the stale block rate, this would increase transaction confirmation times, which is undesirable in EMR medical emergency scenarios.

#### Data On-Chain Versus Off-Chain

Another data architecture trade-off is data on-chain versus off-chain [32]. Smaller data packets on-chain result in smaller storage costs but less data are available on-chain to audit. With a blockchain EMR, storing all patient data on-chain is likely infeasible: the International Data Corporation estimates that medical data will total 2314 exabytes by 2020 [33]. Duplication of data at such scales would be costly for health care providers. Systems that store patient data on-chain also require very strict security. Storing only metadata (ie, access permissions and edit history) is likely to be more suitable. If patient data were to be placed on-chain, unprocessed files should be avoided; raw genomic data, for instance, can be in excess of .1 TB per genome [34].

#### Block Size Versus Throughput

Block size versus throughput [20] is another data architecture trade-off. A blockchain network’s maximum transaction throughput is calculated as maximum block size (ie, how many transactions can be stored in a block) divided by block window. Thus, a large block size is desired. If the maximum block size is too small, there is also a risk that many transactions will not be processed, as blocks will not have enough space to store all the transaction requests being made on the network. However, making the maximum block size too large will inadvertently reduce network throughput, as it will take longer to transmit blocks across a network for mining and validation. Both ends of this trade-off can delay access to EMRs in emergency situations. To maximize the throughput in a network, simulations should be run with different maximum block sizes, as per the study by Xin et al [20], to identify an optimum size. An adaptive block size may also be considered: at times of the day where there are high volumes of transactions, block size can be increased to prevent rejection of transaction requests. Outside of this period, block size can be reduced to increase network throughput. An adaptive system like this is relevant to medicine: not all types of professionals are active 24/7, which may result in times of the day where transaction requests become less frequent.

#### Read and Write Performance Versus Scalability

An additional data architecture trade-off is read and write performance versus scalability [19,22]. Blockchains have different options for holding edit data: data can be held in random access memory (RAM) or nonvolatile memory. RAM is generally faster to access than nonvolatile memory but has less space. Often on blockchains, recent edits are stored in RAM while remaining data are stored on a hard disk. Reducing the number of recent edits stored in RAM reduces read and write performance, delaying updates to the blockchain. However, it allows more data to be stored on nonvolatile memory, making systems more scalable. Given the likely growth in the size of health care data [33], ensuring scalability of blockchain EMRs should be a priority.

#### Method of Information Storage: Scalability Versus Immutability

One final data architecture trade-off is method of information storage: scalability versus immutability [35]. There are different ways to store data on blockchains. Three possibilities to do this for EMRs are as follows: (1) Mirroring: information is converted into hashes, and the hashes are stored on-chain; (2) Digital records: unhashed human-readable information is stored directly on-chain; and (3) Tokenization: information is treated as a token with value, and tokens are exchanged like currency. Mirroring EMRs (eg, those in use by the Estonian government [36]) are space efficient (ie, full EMRs are converted into single hash values) and can show when records stored off-chain have been tampered with, as hashes will no longer match. However, mirroring does not protect the original records through decentralized storage. Digital records guarantee immutability but are less space-efficient than mirroring. Tokenization also permits immutability, but it assumes that data is principally transactional in nature. This may not be true of many situations relevant to EMRs.

### Application Architecture Trade-Offs

#### Expressibility in Blockchain Language

One application architecture trade-off is expressibility in blockchain language [19,22]. Bitcoin’s scripting language has low functionality, making it difficult to exploit. Ethereum instead uses a Turing-complete scripting language (ie, it can compute anything that is computable given enough resources). While this increases versatility, flawed code can be submitted on Ethereum. An example was Ethereum’s Decentralized Autonomous Organization (DAO): designed to operate as an investment fund, an exploit was discovered that allowed hackers to steal US $50,000,000 from the DAO [37]. A blockchain EMR should consider using a low-functionality language to avoid security being compromised by faulty code. However, the language should ideally be expressive enough to permit new types of hospital data to be integrated and to allow for smart contracts to be established.

#### Errors on the Blockchain: Filtering and Expiration

Another application architecture trade-off includes errors on the blockchain: filtering and expiration [38]. Instead of limiting script functionality to stop bugs, automated auditing could be used to identify bugs. However, automated auditing cannot guarantee that bugs will not end up on the chain and could increase network latency. A fail-safe mechanism is to give scripts expiration dates (ie, they will stop working after a certain amount of time). However, this could create confusion in EMR systems, with access to medical data being lost at critical moments.

#### Confirmation Blocks: Confidence Versus Speed

An additional application architecture trade-off includes confirmation blocks: confidence versus speed [21]. Honest and malicious miners may at the same time be working on different blocks to add to the blockchain. The probability of malicious miners creating a long *branch* consisting of multiple blocks is typically low. For this reason, blockchains often include *confirmation blocks* as a safety feature: a fixed number of confirmed blocks must proceed with a block in the chain before its contained transactions are performed. This causes latency between when a block is mined and when a transaction is performed. If more confirmation blocks are required, it reduces the chance of malicious transactions being processed but increases transaction latency. In a medical emergency, this delay could be critical. This trade-off can be adaptively managed; certain requests could require fewer confirmation blocks, allowing for emergency access to essential information.

#### Zero-Knowledge Proof: Security Versus Scalability

Zero-knowledge proof: security versus scalability [39] is another application architecture trade-off. Zerocoin is a blockchain that uses zero-knowledge proof (ZKP). ZKP hides a transaction’s origin, destination, and content, but still allows transfers to be immutable. For EMRs, ZKP permits enhanced privacy; patients would be able to confirm EMR details without revealing that their EMR was queried on the blockchain. This potentially allows for sensitive exchanges to occur on public blockchains. While auditability is a concern for ZKP, Naganuma et al [39] describe a mechanism that permits designated auditors to audit information from ZKP transactions while preventing others from obtaining it. ZKP’s disadvantage is its computational intensity: ZKP requires multiple rounds of communication between the sender and the receiver, with the certainty of truth increasing with every round of communication. This increases network delay and reduces scalability.

#### Blockchain Filtering: Safety Versus Flexibility

Another application architecture trade-off is blockchain filtering: safety versus flexibility [24]. Misconduct occurs on blockchains: Bitcoin contains links to pornography on its immutable ledger [40] and INTERPOL notes that chain updates may be used to inject malware [41]. Avoiding misconduct is essential on a blockchain EMR. One solution is to set a size limit on content added to the chain, as malware and pornography typically require large data packets. However, this could severely limit EMR functionality. An alternative is a filter system, but this could be circumvented and false positives may also occur, preventing the submission of medical data. While using human auditors can ensure accuracy, it could create a human bottleneck and could be used to attack the blockchain. To prevent misconduct, a combination of measures is likely to be the best solution.

#### Consensus: Byzantine Fault Tolerance Versus Non-Byzantine Fault Tolerance

Consensus: Byzantine Fault Tolerance (BFT) versus non-BFT [42] is another application architecture trade-off. A network of actors making a consensus decision is considered *Byzantine Fault Tolerant* if it can achieve consensus when some dishonest actors are present. Two of the properties that define BFT are *Agreement* (ie, all honest actors choose the same block) and *Termination* (ie, all honest actors eventually choose a block). The internet is an asynchronous network (ie, there is no guarantee that a message is delivered in a known time period). In asynchronous networks, BFT is mathematically proven to be impossible [43]: agreement and termination cannot be simultaneously guaranteed. Consensus protocols that require agreement between all honest actors may never terminate. In networks with only a few nodes, *weak* synchrony can be assumed and algorithms such as Practical Byzantine Fault Tolerance (PBFT) [44] allow consensus in these systems. However, PBFT fails in large networks as it requires heavy internode communication. Typical blockchain consensus algorithms (eg, PoW and proof of stake) instead use randomization systems (eg, mining a random nonce) to probabilistically approximate BFT. Such systems guarantee that transactions are processed, but transactions submitted by dishonest actors can be validated. On a blockchain EMR, this could result in data being obtained by malicious actors.

#### Consensus: Agreement Versus Termination

An additional application architecture trade-off is consensus: agreement versus termination [42]. For systems using probabilistic consensus, a trade-off can be made between agreement and termination; applications can have a *time-out* period, after which they consider a transaction to be valid even if full blockchain agreement has not yet been reached. Extending the time-out period delays transaction validation but results in fewer invalid transactions being processed.

#### Transparency Versus Privacy

A final application architecture trade-off is transparency versus privacy [45]. Bhaskaran et al make the case that blockchains involve trade-offs between preserving privacy and promoting transparency. While privacy is paramount in EMRs, there are situations where transparency should be considered. An example of this trade-off would be in verification: if a patient verifies their patient status on the blockchain with a provider, such verification could be shared with other health care providers to avoid a patient repeating the process. This transparency can improve efficiency, but information linking the patient to the original provider sacrifices privacy. While Bhaskaran et al propose a solution to this specific problem that makes use of smart contracts [45], there are more general considerations to be made about when transparency that benefits providers may create potential privacy issues for patients.

### Business Architecture Trade-Offs

#### Control Versus Points of Failure

One business architecture trade-off is control versus points of failure [29]. Blockchain consensus can be determined by different actors. Options include the following: public (ie, consensus is determined by all network nodes), private (ie, a single organization manages blockchain updates), and consortium (ie, blockchain updates are determined by preselected validator nodes). In these systems, there is a trade-off between control and points of failure; in a private blockchain, it is easy to change settings, but a single point of failure means that if the organization is compromised, so is the blockchain. At the other extreme, a public blockchain has consensus verified by the entire network, but changing network properties requires all users to migrate to a new system. Consortium systems are an intermediate: these may be the most practical for EMRs, as there are multiple trusted authorities and businesses that could act as these preselected validator nodes (eg, hospital groups and health insurance companies).

#### Fraction of Consortium: Time Versus Trust

Another business architecture trade-off is fraction of consortium: time versus trust [29]. Alhadhrami et al suggest a hypothetical model where a fraction of validator nodes in a consortium blockchain must sign a transaction for it to be accepted. For an EMR consortium blockchain, choices should be made about the number of nodes that are needed to sign a transaction for it to be validated. More nodes verifying a transaction increases trustworthiness but results in longer verification times. In emergency medical situations, this delay could be critical. If the organizations involved in the consortium are seen to have high security, the fraction of consortium required can be kept low. Furthermore, the fraction of consortium required can be adaptively managed based on how time-critical the patient data are and the nature of the data being requested.

## Discussion

### Principal Findings

At the time of writing, this was the first manuscript identified in literature searches that systematically reviews trade-offs in blockchain design that are relevant to EMRs. While evidence was chosen based on relevance to EMRs, the trade-offs discovered are relevant to blockchain design in other disciplines and industries. Trade-offs were demonstrated with mathematical proofs, real-world examples, and simulations of blockchain networks. Some trade-offs were not directly evidenced, but these trade-offs were logical and comparable to real-world examples. While the list of trade-offs discussed is likely to be nonexhaustive due to study limitations, the trade-offs discovered can improve understanding of blockchain systems for medical professionals, while also providing useful design information for organizations looking to develop blockchain EMRs.

### Limitations

Limitations have been divided into three types, as discussed below.

#### Unresolvable Issues

Firstly, non-English studies were excluded from the review. Secondly, as very few papers discuss the same trade-offs, there is little interpaper support for findings. Thirdly, while most papers contained logical proofs, not all papers contained simulation data. Thus, not all mathematical relationships in trade-offs are known.

#### Issues With Tools

Firstly, a lack of accepted MeSH terms for “design” and “trade-off” meant that alternative keyword terms were chosen nonsystematically. This may have caused exclusion of relevant literature. This is particularly pertinent given that not having “design” in the abstract was used to remove 1076 papers from the literature search. Subsequent reviews should aim to filter through these 1076 papers to see if other pertinent terms can be used to filter papers more effectively. Secondly, While the TOGAF was practical for exploring blockchains, it was not designed for this task and was designed in a preblockchain era. A more suitable framework specifically designed for blockchain analysis may be in existence but was not found for this study. Finally, for most papers, ROBINS-I was an inappropriate tool, as no experimental data was present. Papers instead made claims using logical and mathematical proofs. Furthermore, ROBINS-I was a tool developed for assessing bias in trials involving biological organisms subject to variation. ROBINS-I test questions on topics such as time-varying confounding and participant adherence were not appropriate for assessing bias in computer simulations. Questions relating to deviations from intended interventions were entirely irrelevant, due to the instantaneous nature of simulations. The CASP qualitative tool was used in instances where ROBINS-I was completely unsuited, but the tool is significantly less comprehensive than ROBINS-I.

#### Methodological Issues

Firstly, the choice to research trade-offs in blockchain design rather than potential solutions to trade-offs may have resulted in some irrelevant trade-offs being discussed in the study. Secondly, four papers that may have contained relevant information were excluded due to the level of technicality being too complex. This may have led to relevant trade-offs being excluded. Thirdly, only papers written after December 2016 were included in order to remove papers with outdated information. However, this cutoff year was relatively arbitrary and may have eliminated potentially useful pre-2017 studies. Fourthly, in paper filtering, exclusion criteria 7, 8, and 9 (see Table 2) were relatively arbitrary. They did not remove significant numbers of papers from the search and may have removed useful papers that could have been added to the final review. This is particularly true of criterion 9: papers with financial information may have discussed trade-offs at the business level of enterprise architecture in blockchain design. Finally, due to time constraints, following peer review, only articles in the second database search published before the initial cutoff date (ie, June 15, 2018) were selected to be added to the qualitative synthesis. Identified articles published after the cutoff date should be addressed in a subsequent systematic review, along with an analysis of their potential biases.

### Conclusions

There were three key findings. Firstly, multiple trade-offs can be adaptively managed. These trade-offs include block size, the number of confirmation blocks required for a transaction, and the fraction of consortium required to process transactions in consortium blockchains. On a blockchain, multiple rule sets for exchanging data can be used. This will allow for adaptive management and maximization of the utility of blockchain EMRs.

Secondly, multiple trade-offs involve improving blockchain systems at the cost of security. Given this, data protection standards will be an important factor in determining the effectiveness of a blockchain EMR system. Given these trade-offs, research should be conducted on the potential effects of current data protection laws on blockchain EMRs. For instance, it may be necessary for further legislative development to allow for the effective development of NHS blockchain EMRs.

Finally, scalability can be encountered at one end of a trade-off. Blockchain scalability can be improved by moving data off-chain, using hash mirroring instead of digital records, and not using ZKP, among other methods. More infrastructure investment would be required to improve scalability otherwise. Given this, it is important that providers wishing to implement blockchain EMR systems understand the current and future scale of their institution. This will allow for the appropriate scalability trade-offs to be made.

### A Model National Health Service Blockchain

This report would make the following specific recommendations for a hypothetical NHS blockchain:

1. Blockchains should not interfere with the Local Health and Care Record programs. Instead, blockchains can be used to manage long character (LCHR) metadata and access permissions for off-chain LCHR EMRs.
2. Given that there are multiple trusted institutions that form part of the NHS structure, a consortium blockchain managed by multiple NHS institutions should be considered. Such a system could potentially make use of PBFT.
3. The blockchain should have an adaptive block size, potentially implemented by machine learning.
4. A blockchain should be built with a scripting language that is specific to NHS needs, one that is difficult to exploit and minimizes the risk of insertion of potentially hazardous data like malware onto the blockchain. Expiration dates on scripts should also be mandated to prevent exploits being permanent.
5. A small number of confirmation blocks should be required for read-only access to patient basic emergency medical data (eg, allergies). More conformation blocks should be required for full access and edit permissions.

### Recommendations for Future Research

Future studies should aim to quantify the mathematical relationships between all identified trade-offs in blockchain design. If this is done comprehensively, this will allow for the exact parameters of a blockchain to be purposely selected when designing a blockchain EMR for a specific provider. Identified trade-offs should also ideally be assessed with data from real-world blockchain EMRs. This will allow the impacts of the trade-offs discussed in this paper to be assessed. In addition, the impact of data protection legislation on blockchain systems should be investigated. The security requirements of health care providers may mean that large trade-offs must be made. The impacts of such trade-offs must be fully understood before a health care provider adopts a blockchain EMR. Given study limitations, further literature searches should be performed to find more examples of blockchain design trade-offs. Discovering more trade-offs that can be adaptively managed and discovering how the scalability of blockchains can be increased through trade-offs will help maximize the utility of blockchain-based EMRs.

## Appendix

Multimedia Appendix 1 Exact search strings used in databases.

Multimedia Appendix 2 Review of paper quality using the Risk Of Bias In Non-randomized Studies—of Interventions (ROBINS-I) checklist.

Multimedia Appendix 3 Critical Assessment Skills Programme (CASP) analysis of papers.

## Abbreviations

BFT: Byzantine Fault Tolerance
CASP: Critical Assessment Skills Programme
DAO: Decentralized Autonomous Organization
EMR: electronic medical record
IEEE: Institute of Electrical and Electronics Engineers
IoT: internet of things
LCHR: long character
MeSH: Medical Subject Headings
NHS: National Health Service
PBFT: Practical Byzantine Fault Tolerance
PICO: Population, Intervention, Comparison, and Outcome
PoW: proof of work
PRISMA: Preferred Reporting Items for Systematic Reviews and Meta-Analyses
RAM: random access memory
ROBINS-I: Risk Of Bias In Non-randomized Studies—of Interventions
SENS: Strategies for Engineered Negligible Senescence
TOGAF: The Open Group Architecture Framework
ZKP: zero-knowledge proof
