# Supplementary figures and images for: Design Choices and Trade-Offs in Health Care Blockchain Implementations: Systematic Review
Source: J Med Internet Res. 2019 May 10;21(5):e12426. doi: 10.2196/12426 (PMC6533871; doi:10.2196/12426)

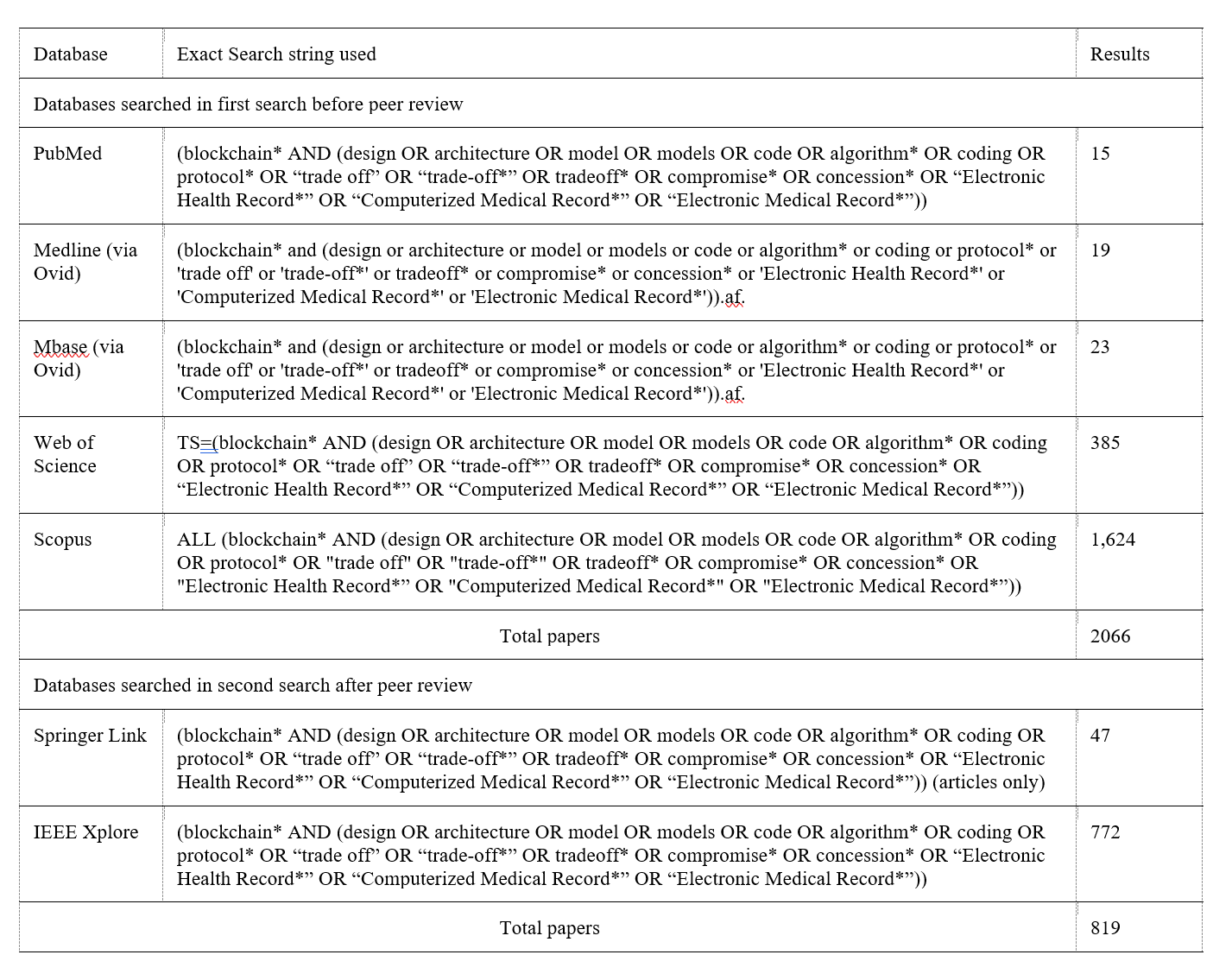

Supplement: Multimedia Appendix 1 [file jmir_v21i5e12426_app1.png]

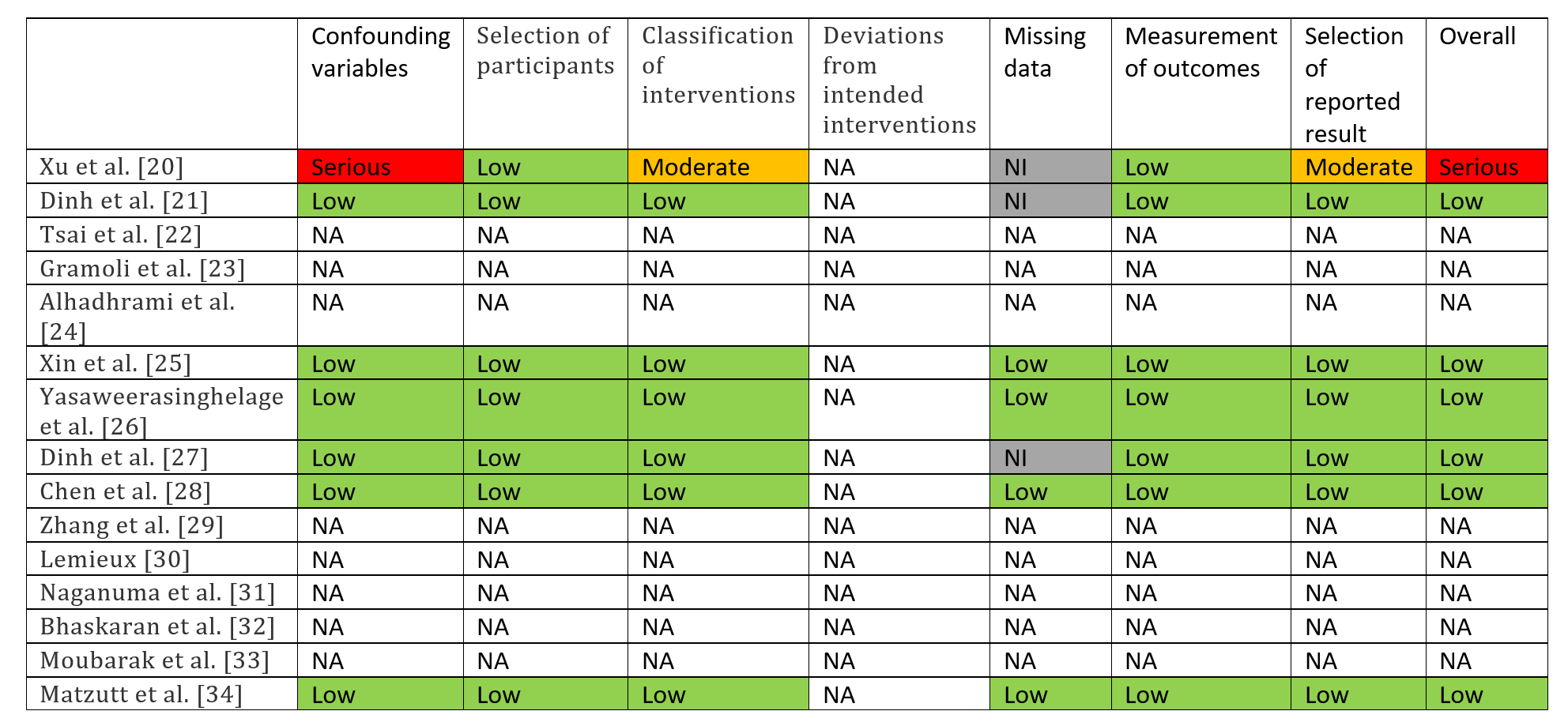

Supplement: Multimedia Appendix 2 [file jmir_v21i5e12426_app2.png]

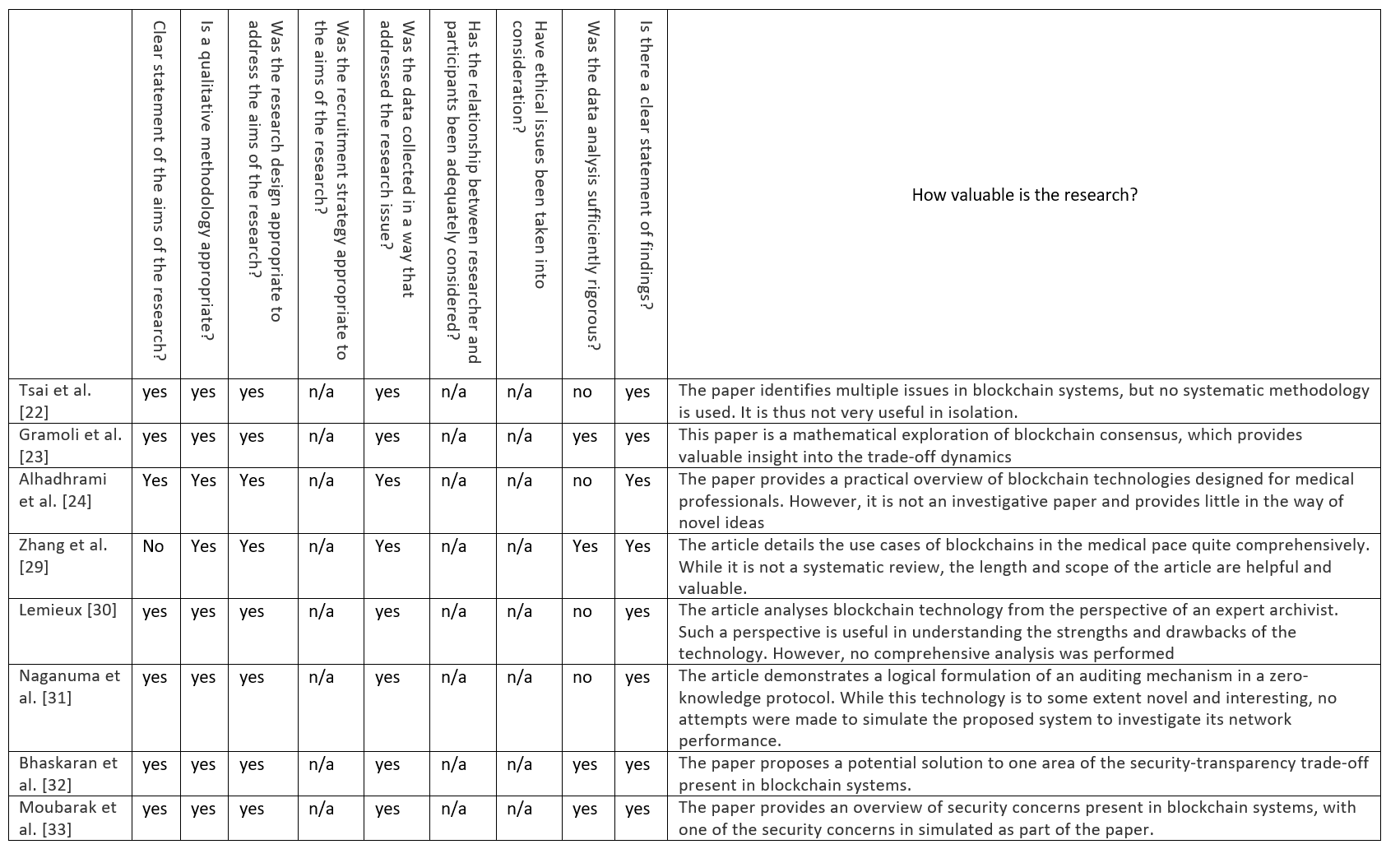

Supplement: Multimedia Appendix 3 [file jmir_v21i5e12426_app3.PNG]
